# Impact of Diabetic Stress Conditions on Renal Cell Metabolome

**DOI:** 10.3390/cells8101141

**Published:** 2019-09-24

**Authors:** Simon Lagies, Roman Pichler, Tillmann Bork, Michael M. Kaminski, Kevin Troendle, Stefan Zimmermann, Tobias B. Huber, Gerd Walz, Soeren S. Lienkamp, Bernd Kammerer

**Affiliations:** 1 Center for Biological Systems Analysis (ZBSA), Albert-Ludwigs-University Freiburg, Habsburgerstr. 49, 79104 Freiburg, Germany; 2 Spemann Graduate School of Biology and Medicine (SGBM), University of Freiburg, 79104 Freiburg, Germany; 3 Faculty of Biology, University of Freiburg, 79104 Freiburg, Germany; 4 Department of Medicine, Renal Division, Medical Center–University of Freiburg, Faculty of Medicine, University of Freiburg, 79106 Freiburg, Germany; 5 Laboratory for MEMS Applications, IMTEK–Department of Microsystems Engineering, University of Freiburg, 79110 Freiburg, Germany; 6 III. Department of Medicine, University Medical Center Hamburg-Eppendorf, 20246 Hamburg, Germany; 7 BIOSS Centre of Biological Signalling Studies, University of Freiburg, 79104 Freiburg, Germany; 8 Institute of Anatomy, University of Zurich, 8057 Zurich, Switzerland

**Keywords:** diabetic kidney disease, metabolomics, GC-MS, diabetic nephropathy, albumin stress, tubule, podocyte, diabetic complication, polyol metabolism

## Abstract

Diabetic kidney disease is a major complication in diabetes mellitus, and the most common reason for end-stage renal disease. Patients suffering from diabetes mellitus encounter glomerular damage by basement membrane thickening, and develop albuminuria. Subsequently, albuminuria can deteriorate the tubular function and impair the renal outcome. The impact of diabetic stress conditions on the metabolome was investigated by untargeted gas chromatography–mass spectrometry (GC-MS) analyses. The results were validated by qPCR analyses. In total, four cell lines were tested, representing the glomerulus, proximal nephron tubule, and collecting duct. Both murine and human cell lines were used. In podocytes, proximal tubular and collecting duct cells, high glucose concentrations led to global metabolic alterations in amino acid metabolism and the polyol pathway. Albumin overload led to the further activation of the latter pathway in human proximal tubular cells. In the proximal tubular cells, aldo-keto reductase was concordantly increased by glucose, and partially increased by albumin overload. Here, the combinatorial impact of two stressful agents in diabetes on the metabolome of kidney cells was investigated, revealing effects of glucose and albumin on polyol metabolism in human proximal tubular cells. This study shows the importance of including highly concentrated albumin in in vitro studies for mimicking diabetic kidney disease.

## 1. Introduction

During the last decades, diabetes mellitus has become a major socio-economic burden for health care systems and societies all over the world. In 2017, 425 million people were affected by diabetes worldwide, and the prevalence of the disease in adults rose to 12.2% in Germany [1]. The growing incidence and prevalence have led to immense challenges for clinicians and caretakers. Diabetes results in tremendous suffering, reduced quality of life, and significant co-morbidities like cardiovascular disease, stroke, retinopathy, peripheral neuropathy, and peripheral artery occlusive disease [2,3]. In particular, the kidney is susceptible to the microvascular complications of diabetes, and diabetic kidney disease (DKD) represents a major cause of morbidity and mortality in diabetic patients [4]. DKD occurs in 20%–40% of type 2 diabetic patients, and induces a progressive decline of the glomerular filtration rate (GFR), leading to end-stage renal disease [5]. It is the most common cause of renal replacement therapy, accounting for over 40% of new cases [6]. The leading clinical manifestations of diabetic nephropathy are progressive chronic kidney disease and albuminuria [7].

Diabetes mellitus is a complex metabolic disease. In DKD, specific alterations of the metabolic pathways occur, like an upregulation of glycolysis, tricarboxylic acid cycle (TCA cycle), and β-oxidation [8]. Concurrently, several studies showed mitochondrial dysfunction and reduced mitochondrial biogenesis [8,9]. It has long been known that the polyol pathway plays an important role in the pathogenesis of diabetic kidney disease [10]. In conditions of hyperglycemia, when glucose phosphorylation by hexokinase is saturated, up to 30% of the glucose turnover can be metabolized in the polyol pathway [11,12]. Aldo-keto reductase (AR) is the rate-limiting enzyme of the polyol pathway, and catalyzes the reduction of glucose to sorbitol [12]. Transgenic mice overexpressing AR showed an aggravation of kidney complications under diabetic conditions [13]. The second reaction of the polyol pathway is catalyzed by sorbitol dehydrogenase, which converts sorbitol to fructose. This pathway contributes to the redox imbalance in diabetes, which, in turn, can lead to oxidative stress [14,15,16]. Interestingly, the accumulation of fructose can elicit tubulointerstitial inflammation and renal injury [17].

In a recently published metabolomics study, Bernardo-Bermejo et al. analyzed glucose-induced changes of exo- and endo-metabolome in a human proximal tubular cell line (HK-2 cells), using an untargeted metabolomic strategy based on liquid chromatography–mass spectrometry [18]. HK-2 cells were exposed to different glucose concentrations and were analyzed by the application of two chromatographic approaches (hydrophilic interaction and reversed-phase liquid chromatography). The group could detect several altered metabolic pathways, including nitrogenous bases metabolism, amino acid metabolism, and polyol metabolism. Showing elevated concentrations of sorbitol, they could find evidence for the hyperglycemia-induced activation of the polyol pathway.

Renal proximal tubule epithelial cells/telomerase reverse transcriptase 1 (RPTEC/TERT1) cells are a human proximal tubular cell line widely used for studies of the proximal tubule [19,20]. After the immortalization of primary human proximal cells with hTERT, the RPTEC/TERT1 cells maintain many characteristics of proximal tubular cells in vivo, remain highly differentiated, and form tight epithelial layers [19]. The expression of specific transporters like the cubilin and megalin (LRP2) receptors, which mediate the endocytosis of albumin [21], suggests that this cell line is highly suitable for studying the implications of diabetic kidney disease and albuminuria.

Previously, we described the direct conversion of fibroblasts to induced renal epithelial tubular cells (iRECs) by lentiviral transduction of the four transcription factors Pax8, Hnf1b, Hnf4a, and Emx2 [22]. iRECs represent a tubular cell line and bear a substantial similarity to primary tubular cells, as shown by transcriptional profiling and CellNet [23]-based characterization [22]. Analogously to the RPTEC/TERT1 cells, iRECs express proximal-tubule specific transporters like Oct2 (organic cation transporter-2; Slc22a2) and the apolipoprotein-receptor megalin (Lrp2). Moreover, the detection of microvilli and evidence for endocytotic uptake of albumin emphasizes the applicability of iRECs for studies of the proximal nephron tubule. In a recent study [24], we analyzed the metabolome of iRECs by gas chromatography–mass spectrometry (GC-MS), showing a high resemblance to mouse kidneys. Interestingly, treatment of iRECs with the nephrotoxic chemotherapeutic drug cisplatin led to characteristic changes of the endo- and exo-metabolome [24], which can also be detected in in vivo studies.

The mIMCD-3 cell line represents the murine inner medullary collecting duct, and was immortalized by SV40. mIMCD-3 cells display typical characteristics of epithelial cells of the inner medullary collecting duct, such as the expression of the amiloride-sensitive epithelial sodium channel or the N-methyl-D-aspartate receptor [25,26].

In 2002, the lab of Peter Mundel developed a human podocyte cell line, which was immortalized by a temperature sensitive *SV40-T* gene. At 33 °C, these podocytes proliferate in an undifferentiated state under the control of SV40, but at 37 °C, when the expression of SV40 is terminated, they differentiate and express specific podocyte markers such as nephrin, podocin, synaptopodin, or p57 [27]. This cell line represents a commonly- and widely-used in vitro system to study specific questions concerning podocytes and the glomerulus.

Although the tubular system and distal parts of the nephron are increasingly in the focus of DKD research, the impact of highly concentrated albumin on the tubular epithelial cell metabolism has not been analyzed in in vitro studies yet. We speculated that this stressful factor, in combination with elevated glucose levels, affects the renal cell metabolism. In this study, we report for the first time an untargeted metabolomics approach to reveal the effects of albumin and glucose on different renal cell lines.

## 2. Materials and Methods

### 2.1. Cell Culture

The human immortalized podocytes were kindly provided by Moin Saleem (Bristol, U.K.) and were cultured as reported [27]. In brief, the cells were cultured in RPMI 1640, including 10% fetal calf serum (FCS), penicillin/streptomycin, and insulin/transferrin/selenite (Roche), as well as pyruvic acid, minimal essential medium, and HEPES buffer (Life technologies). After 10–14 days at 37 °C, differentiation was induced. Afterwards, the cells were incubated with the indicated glucose concentration for 24 h.

Mouse embryonic fibroblasts (MEFs) were reprogrammed to iRECs as previously described [22]. In short, Ksp-Cre (kidney-specific protein; Cdh16) reporter MEFs, containing a Tomato/GFP cassette, were obtained from the limbs of E13.5 mouse embryos and kept in MEF medium (MEFM), containing Dulbeccos’s modified Eagle’s medium (DMEM, Gibco™), 2 mM L-glutamine, penicillin/streptomycin, and 10% fetal bovine serum (FBS). At confluency, the cells were split 1:4 using 0.25% trypsin-EDTA, and transduced with four lentiviruses, each containing one of the murine transcription factors mPax8, mHnf1b, mEmx2, and mHnf4a. The transcription factors were cloned into the pWPXLd lentiviral vector (Addgene no. 12258). The concentrated lentiviruses were diluted 1:100 to 1:1000 in MEFM containing 8 µg mL^−1^ polybrene (sc-134220), and were transduced for 12 h for seven consecutive days. In the MEFs, which were efficiently reprogrammed to iRECs, the expression of a Cre recombinase under the control of the Ksp promoter leads to a change in fluorescence from Tomato to GFP. Consecutively, GFP-positive iRECs were sorted 14 days after the last lentiviral transduction, using a BD FACSAria™ Fusion flow cytometer (Becton Dickinson).

RPTEC/TERT1 cells (ATCC^®^ CRL-4031™) were purchased from ATCC, Manassas, VA, USA. These cells represent an immortalized cell line after the retroviral transduction of pLXSN-hTERT. They were grown in Renal Epithelial Cell Growth Medium (REGM™; Lonza, CC-3190), containing REBM™ Basal Medium (CC-3191) and REGM™ SingleQuots™ supplements (CC-4127).

The iRECs and mIMCD-3 cells (ATCC^®^ CRL-2123™, ATCC, Manassas, VA, USA) were cryoconserved in freezing medium, containing 10% DMSO, 40% FBS, and 50% MEFM, and stored in liquid nitrogen. RPTEC/TERT1 cells were cryoconserved in freezing medium containing 50% DMEM/F12 (Gibco™). After thawing, all of the cell lines were seeded with an initial density of 1 · 10^6^ cells per 10 cm dish, and were grown until confluency.

For metabolomic and transcriptional analyses, the cell lines were cultivated in the following four different media conditions for 48 h: high glucose (HG), high glucose plus albumin (HG+Alb), normal glucose (NG), and normal glucose plus albumin (NG+Alb). The following media were applied, containing two different glucose concentrations, namely: DMEM high glucose (4.5 g L^−1^ glucose (24.98 mM, 450 mg dL^−1^), Gibco™ 41966029) and DMEM low (Normal) glucose (1.0 g L^−1^ glucose (5.55 mM, 100 mg dL^−1^), Gibco™ 31885023). For albumin overload, sterile filtered bovine serum albumin was added to a final concentration of 10 mg mL^−1^ (Bovine Serum Albumin (BSA) Fraction V, CAS 9048-46-8, Biomol). Penicillin/streptomycin and FBS (10% final concentration FBS) were added to each media composition. For the metabolomic analysis, four replicates per media condition were cultivated. After harvesting, the cells were directly processed for metabolite extraction or RNA extraction.

### 2.2. Quantitative RT-PCR

The total RNA was extracted with the QIAzol Lysis Reagent (Qiagen no. 79306), and was isolated using the RNeasy Plus Universal Mini Kit (Qiagen no. 73404). The QuantiTect Reverse Transcription Kit (Qiagen no. 205311) was applied to reversely transcribe 1 µg of total RNA to cDNA, according to the manufacturer’s instructions. Quantitative PCR was performed on a Roche LightCycler^®^ 480 instrument using 10 ng of cDNA, gene-specific primers, and Takyon™ SYBR^®^ Master Mix (Takyon™ No Rox SYBR^®^ MasterMix dTTP Blue, Eurogentec, UF-NSMT-B0701). The primers are listed in Supplementary Table S1. For the normalization of threshold cycle (CT) values, primers aligning the following housekeeping genes were used: Tbp (mouse) and glyceraldehyde-3-phosphate dehydrogenase (GAPDH; human). The qPCR data were analyzed applying the comparative CT method [28]. Normalized values were statistically compared using an unpaired Student’s *t*-test.

### 2.3. Untargeted Metabolomics

Untargeted metabolomics was conducted as previously reported [24]. In brief, the cells were washed with 0.9% NaCl (Sigma Aldrich) and harvested with ice-cold methanol:water 9:1 (*v*:*v*) by scraping. After mechanical lysis and centrifugation by a Precellys24 (Bertin), the metabolite containing supernatant was evaporated. After derivatization with methoxyamine hydrochloride (Supelco) in pyridine (Roth) and N-methyl-N-(trimethylsilyl)trifluoroacetamide (Sigma Aldrich), the samples were analyzed by GC-MS with an HP5-MS column (60 m × 0.25 mm × 0.25 µm). The MS was calibrated with perfluorotributylamine prior to each sequence. An alkane mixture with even carbon numbers was measured before the biological analysis in order to obtain system-independent retention indices. The system equilibration was performed by the injection of several pooled samples, which were regularly monitored within the randomized sample sequences. The consistently found metabolites [29] were identified by spectra and retention index matching to different libraries [30], including an in-house database. To allow for a better comparability between the different cell lines, all metabolites were, in addition to internal standard and peak sum normalization, normalized to the corresponding normal glucose condition so as to obtain fold-changes. Statistical analyses were carried out by MetaboAnalyst 4.0 [31]. Analysis of variance (ANOVA) with the following FDR(false discovery rate)-adjustment was carried out in order to determine significance (FDR-adjusted q-value of <0.05). The pairs of significance were revealed by Tukey’s honestly significant difference (HSD) post-hoc test. The results of the latter statistical analyses are depicted in Supplementary Table S2. For principal component analysis and heat map generation, the values were range-scaled.

### 2.4. Data Availability

The data set of this study will be made publicly available in MetaboLights, under the study identifier MTBLS1093 (https://www.ebi.ac.uk/metabolights/MTBLS1093).

## 3. Results and Discussion

To simulate diabetic complications in kidney cells in vitro, we applied three different glucose concentrations (5, 11, and 30 mM) to undifferentiated and differentiated podocytes, which are the main epithelial cells in the glomerulus and build up the slit diaphragm. Moreover, we applied two glucose concentrations (5.5 and 25 mM) to two tubular cell lines (RPTECs/TERT1 and iRECs) and a collecting duct cell line (mIMCD-3), in order to simulate hyperglycemia in this nephron compartment. As a consequence of the glomerular collapse and podocyte foot process effacement, a diffuse leak in the glomerular filtration barrier occurs and serum proteins are excreted into the urine [32], where they can impair tubular function. Microalbuminuria and albumin-induced tubulopathy are increasingly in focus regarding the renal outcome and progression of DKD [33]. Therefore, we additionally exposed all three tubular cell lines to elevated albumin concentrations. We applied an albumin concentration of 10 mg mL^−1^, which has already been used in other albumin overload experiments [34,35].

**Figure 1:**
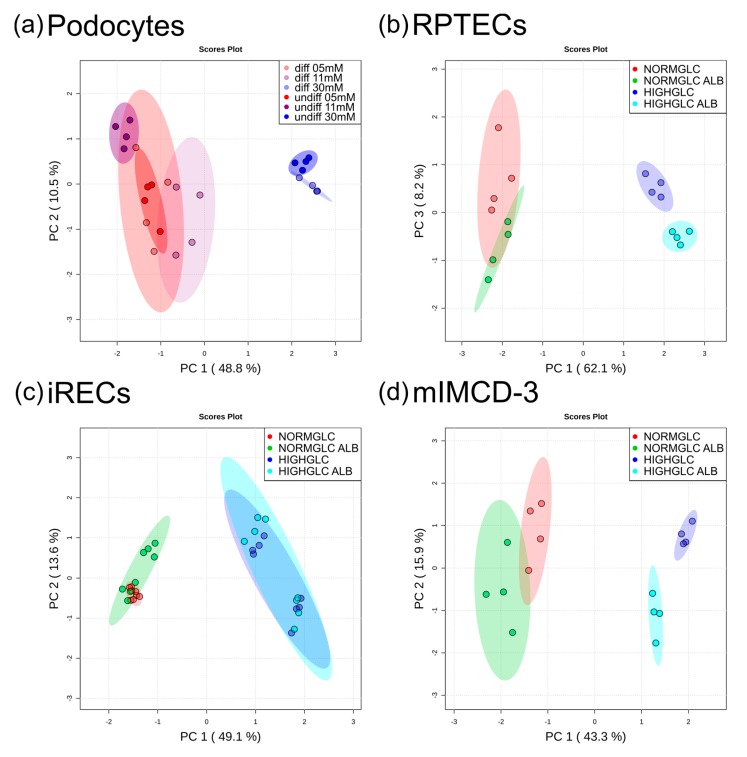
Principal component analysis (PCA) of different cell lines. (**a**) Glomerular cells showed global metabolic alterations upon high glucose treatment. (**b**) Human proximal tubular cells revealed a global alteration between the glucose concentrations, and further by the addition of albumin in the high glucose condition. (**c**) Murine proximal tubular cells were discriminated by the glucose concentration. (**d**) Murine collecting duct cells were separated by the glucose concentration, with further discrimination by albumin addition in the high glucose condition. In each cellular system, that is, differentiated podocytes, undifferentiated podocytes, RPTECs, induced renal epithelial tubular cells (iRECs), and mIMCD-3 cells, the intensities were normalized to the corresponding physiological condition so as to focus on the treatment specific effects (*n* = 4–8).

Untargeted GC-MS analyses revealed global metabolic changes in all of the cell lines analyzed. For the podocytes, we used two growth conditions resulting in either undifferentiated or differentiated podocytes. To focus on the specific impact of glucose on the metabolome, we normalized each condition to the corresponding physiological glucose concentration (5 mM). In both podocyte cultures, a high glucose administration led to 48.8% of total alterations, as documented by principal component (PC) 1 (Figure 1a).

**Figure 2:**
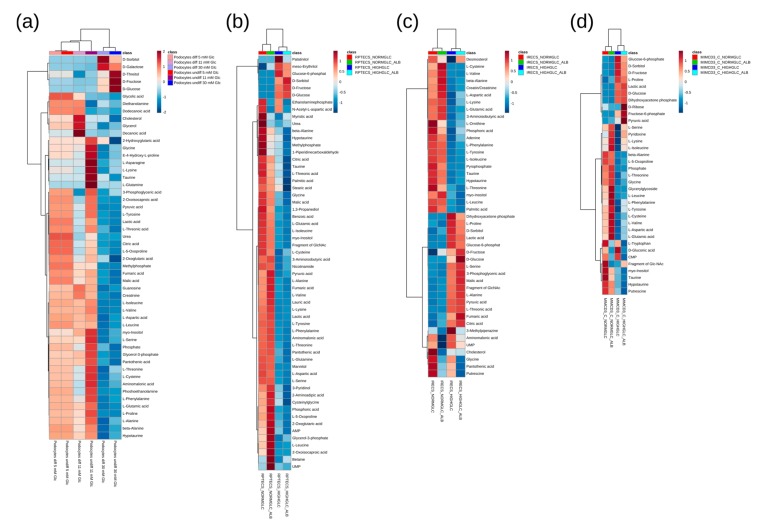
Heat map and cluster analysis (Pearson/Ward) of the significantly altered (FDR corrected *q* < 0.05) metabolites for the podocyte cell lines (**a**), for the proximal tubular cell lines RPTECs (**b**), and iRECs (**c**), as well as for the collecting duct cell line mIMCD-3 (**d**). Range scaling was performed to obtain the z-scores. “Fragment of Glc-NAc” is an indistinguishable fragment of either N-acetylglucosamine-1-phosphate or N-acetylglucosamine-uridine diphosphate, with the first being the direct precursor of the latter in the hexose amine pathway. The individual results and pairs of significance obtained by Tukey’s HSD are displayed in Supplementary Table S2. In each cellular system (i.e., differentiated podocytes, undifferentiated podocytes, RPTECs, iRECs, and mIMCD-3 cells), the intensities were normalized to the corresponding physiological condition so as to focus on the treatment specific effects. *n* = 4–8.

Accordingly, in both of the differentiation states, two main metabolite clusters were observed in the heat map, as follows: in podocytes grown in a highly concentrated glucose environment, the intermediates of the polyol pathway were drastically increased, while amino acids and other organic acids were decreased compared with normal and intermediate glucose levels (Figure 2a).

Correspondingly to podocytes, the samples were normalized to the associated physiological condition (5 mM glucose without albumin stress) in RPTECs, iRECs, and mIMCD-3 cells. In all three of the tubular cell lines, the differences in the glucose concentration had the most prominent effect on the metabolome (Figure 1b–d). In RPTECs, these changes were represented by PC 1 with 62.1% of alterations (Figure 1b). PC 2 accounted for 9.2% of alterations, and reflected the biological variance (see Supplementary Figure S1). PC 3 revealed an impact of albumin overload, leading to 8.2% of total alterations (Figure 1b). Importantly, a discrimination by albumin could only be observed under high glucose conditions. In the RPTECs cultivated in physiological glucose conditions, the addition of excess albumin did not lead to separate clustering in PC 3. In iRECs, the glucose concentration accounted for 49.1% of total changes, and the metabolome was not affected globally by the albumin overload (Figure 1c). In mIMCD-3 cells, the glucose concentration accounted for 43.3% (PC 1), and the albumin treatment for 15.9% (PC 2) of total alterations. Analogously to RPTECs, global differences by the addition of albumin could only be detected under high glucose concentrations in mIMCD-3 cells (Figure 1d).

A heat map analysis of the significantly altered metabolites (all of the statistical results in this study are depicted in Supplementary Table S2) with corresponding cluster analysis (Pearson and Ward) confirmed the results obtained by the principal component analyses (Figure 2). In the high glucose condition of all of the nephron segments, including the collecting duct, most amino acids were downregulated. The ketogenic and glucogenic amino acids were equally reduced. The downregulation of amino acids is in accordance with the results of a GC-MS analysis conducted in the renal cortex of Wistar rats [36]. In these animals, diabetes was induced by streptozotocin treatment, and the diabetic kidneys showed a downregulation of most amino acids. Moreover, in a metabolomics study analyzing the urinary excretion of metabolites in patients with diabetic kidney disease, amino acids, fatty acids, and intermediates of the TCA cycle were reduced in the urine. According to the authors, this downregulation of certain metabolites could be due to an impaired mitochondrial function and reduced mitochondrial biogenesis [9]. In our data, branched-chain amino acids (BCAAs), like valine, leucine, and isoleucine, were reduced in all of the analyzed cell lines (Figure 2). In streptozotocin-treated rats, two weeks after the induction of diabetes, elevated levels of intermediates in the catabolism of BCAAs could be detected [37]. On the other hand, there are studies that claim increased levels of BCAAs in diabetic conditions [8]. Although data on the intracellular levels of BCAAs in diabetic kidney disease are still inconsistent, there is an upregulation of plasma levels of branched-chain and aromatic amino acids in diabetes. This upregulation can even be used as a predictor of future onset of diabetes [38].

**Figure 3:**
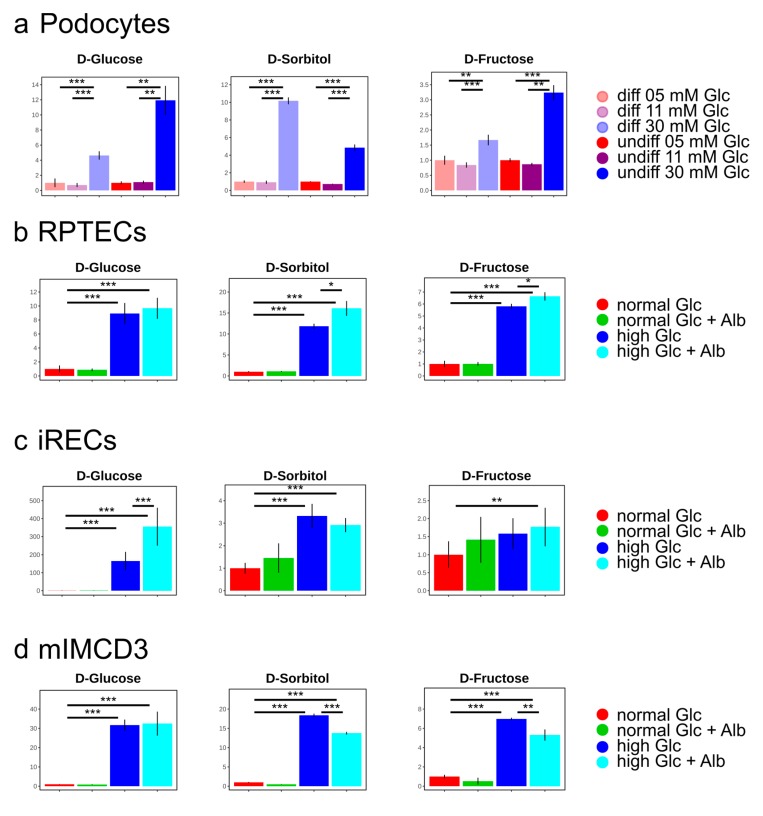
Bar charts of the polyol pathway metabolites for the podocyte cell lines (**a**), for the proximal tubular cell lines RPTECs (**b**), and iRECs (**c**), as well as for the collecting duct cell line mIMCD-3 (**d**). Fold changes to the normal glucose condition. Error bars indicate standard deviation. * *p* <0.05; ** *p* < 0.01; *** *p* < 0.001; *n* = 4–8.

As expected, the concentration of glucose was drastically increased in the high glucose conditions of all of the analyzed cell lines. Moreover, its direct downstream intermediates, including sorbitol and fructose, were also significantly elevated (Figure 2 and Figure 3), indicating an increased glucose metabolization through the polyol pathway in podocytes (Figure 3a), proximal tubular cells (Figure 3b,c), and collecting duct cells (Figure 3d). Interestingly, the addition of highly concentrated albumin led to an additional significant boost of the polyol pathway intermediates in RPTECs (Figure 3b), whereas glucose-6-phosphate was slightly reduced (Figure 2b). This suggests that the albumin uptake could promote enhanced polyol pathway activation under high glucose concentrations. In the other proximal tubular cell line (iRECs), the interplay of high glucose and albumin was necessary to gradually increase fructose, while sorbitol was already elevated by the supraphysiological glucose concentrations (Figure 3c). In mIMCD-3 cells, sorbitol and fructose were also increased by the addition of high glucose levels (Figure 3d). However, in iRECs and mIMCD-3, we could not detect a significant additional increase in the fructose concentration caused by albumin (Figure 3c,d).

In all of the cell lines, we observed a decrease of taurine and myo-inositol after applying high glucose concentrations. In RPTECs, the addition of albumin further lowered the levels of taurine and myo-inositol (Figure 2). A decrease of these two metabolites had already been linked to polyol pathway activation [39], supporting our findings. Among others, these low molecular mass compounds serve as intracellular osmolytes. They are correspondingly downregulated when the concentration of sorbitol increases, in order to maintain osmotic pressure [39]. Moreover, taurine acts as an antioxidant and counteracts the generation of reactive oxygen species (ROS) in diabetes [40].

Taken together, in all three nephron segments (i.e., glomerular, proximal tubular, and collecting duct cells), the supraphysiological glucose levels led to a dramatic increase in sorbitol and fructose concentrations. This effect was aggravated by albumin in RPTECs.

**Figure 4:**
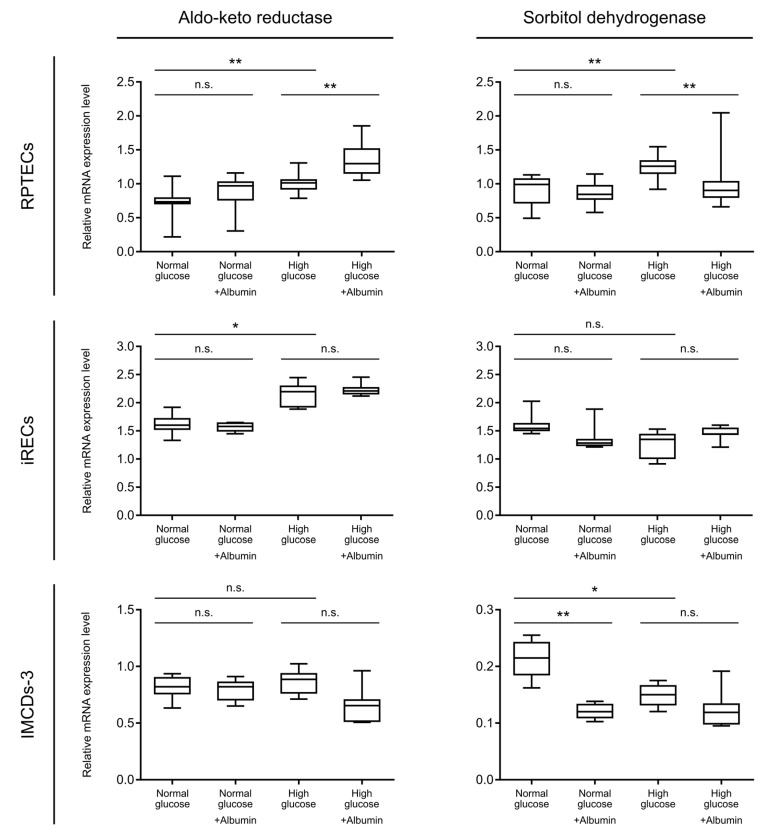
Relative mRNA expression level of aldo-keto reductase and sorbitol dehydrogenase as detected by qPCR analysis. Comparison between normal and high glucose; normal glucose with/without albumin overload, and high glucose with/without albumin overload. Data are shown for RPTECs (upper line), iRECs (intermediate line), and IMCD-3 cells (lower line). Boxplots display the mean values of three replicates with 95% confidence intervals (whiskers). *p*-values: n.s. not significant, * *p* < 0.05, ** *p* < 0.01.

In order to further validate our hypothesis of enhanced metabolization through the polyol pathway under high glucose conditions, we tested the mRNA expression levels of aldo-keto reductase and sorbitol dehydrogenase in the tubular cell lines. Concordantly to the increased sorbitol concentrations, the transcript level of aldo-keto reductase, the rate limiting enzyme of the polyol pathway, was significantly upregulated upon the addition of high glucose in both proximal tubular cell lines, compared with normal glucose (Figure 4, upper and intermediate line). A further increase was observed upon albumin overload in the high glucose condition in RPTECs (Figure 4, upper line), which is in accordance to the additional increase in sorbitol levels. The next enzymatic step, mediated by sorbitol dehydrogenase, was also significantly upregulated under hyperglycemic conditions in RPTECs. Contrary to our expectations, the expression level of sorbitol dehydrogenase upon additional albumin treatment in a high glucose concentration was reduced to normoglycemic levels (Figure 4, upper line). One possible explanation of this phenomenon could be a negative feedback mechanism of increased fructose concentrations on the sorbitol dehydrogenase expression. On the other hand, this inadequate increase in the enzyme levels could further explain the sorbitol accumulation detected in RPTECs in a high glucose and albumin concentration (Figure 3b). For quantitative RT-PCR in RPTECs, GAPDH (glyceraldehyde-3-phosphate dehydrogenase), a known enzyme involved in glycolysis, was used as a housekeeping gene in order to normalize the qPCR values. For internal control, heat shock 90kDa protein 1 beta (HSPCB) was also measured as a housekeeping gene. The expression levels of both genes are not dependent on the glucose or albumin concentrations, as seen in Supplementary Figure S2. In iRECs, high glucose concentrations led to elevated aldo- keto reductase levels, reflecting an elevated sorbitol concentration. In accordance with our metabolomics data, this effect could not be further enhanced by an albumin overload. The expression of sorbitol dehydrogenase, on the other hand, did not show any dependence on the glucose and albumin levels in iRECs (Figure 4, intermediate line). In mIMCD-3 cells, the mRNA expression levels of the aldo-keto reductase were not changed (Figure 4, lower line). In general, sorbitol dehydrogenase was expressed at low levels in mIMCD-3 cells, and also showed an opposing regulation upon treatment with glucose and albumin. Although the sorbitol and fructose concentrations were elevated in mIMCD-3 cells, this could not be explained by an increase in the transcription levels of the enzymes involved. This further supports the hypothesis that the collecting duct reacts differently to diabetic stress conditions than proximal tubular cells (Figure 4). In both nephron compartments, the expression of hexokinase-I was not increased upon diabetic stress exposure (Supplementary Figure S3).

Although the inhibition of aldo-keto reductase seems to be beneficial in diabetes [41], little is known about the regulation of the transcript levels of aldo-keto reductase and sorbitol dehydrogenase in diabetic kidney disease. In a study published in 2001, an upregulation of aldo-keto reductase and sorbitol dehydrogenase mRNA could be detected in patients with diabetes and diabetic kidney disease, depending on the glucose levels [42]. On the contrary, in diabetic neuropathy, the mRNA level is not affected by the glucose levels [43].

Our data clearly support the hypothesis that the polyol pathway is activated in kidney cells under diabetic conditions, and we speculate that the concentrations of metabolites and enzyme transcription levels depend on the nephron segment analyzed. However, we cannot fully substantiate this hypothesis, because the heterogeneity of the analyzed cellular systems regarding the species and immortalization technique certainly biases this conclusion. Although it is a limitation of this study that different metabolic responses detected along the nephron are in part caused by the heterogenic origin of the cell lines, many metabolic alterations certainly arise from segment-specific physiological behavior. The general activation of the polyol pathway and the associated decrease of taurine and myo-inositol are robustly shown, occurring likewise in all cells.

## 4. Conclusions

In this study, we analyzed the impact of diabetic complications on different nephron segments in murine and human cell models, covering the glomerulus, proximal tubule, and collecting duct. We could detect a concomitant activation of the polyol pathway, especially an accumulation of sorbitol, in each cell line tested. Importantly, we could show that the albumin stress further increased this activation in the human proximal tubule. These results could be validated by a qPCR analysis of the rate limiting enzyme in the polyol pathway, the aldo-keto reductase. Furthermore, global differences in the metabolome were elucidated, covering amino acid metabolism and others. These results emphasize the importance of including albumin as a diabetic stress factor for tubular cells when studying diabetic kidney disease in in vitro models.

## Supplementary Materials

The following are available online at https://www.mdpi.com/2073-4409/8/10/1141/s1: Figure S1: Principal component analysis of RPTECs. Figure S2: Ct values of housekeeping genes used for quantitative RT-PCR in RPTECs. Figure S3: Relative mRNA expression of hexokinase 1. Table S1: Primers used in this study [44,45,46]. Table S2: Results of statistical analyses.
