# Supplementary material for: Impact of Diabetic Stress Conditions on Renal Cell Metabolome
Source: Cells. 2019 Sep 24;8(10):1141. doi: 10.3390/cells8101141 (PMC6829414; doi:10.3390/cells8101141)
Supplement: Supplementary file 1 [file cells-08-01141-s001.zip › Revised Supplementary information.pdf]

Impact of diabetic stress conditions on renal cell metabolome – Supplementary material

Simon Lagies[1,2,3+], Roman Pichler[4+], Tillmann Bork[4], Michael M. Kaminski[4], Kevin Troendle[5], Stefan Zimmermann[5], Tobias B. Huber[6], Gerd Walz[4,7], Soeren S. Lienkamp[4,7,8], Bernd Kammerer[1,7*]

[1] Center for Biological Systems Analysis (ZBSA), Albert-Ludwigs-University Freiburg, Habsburgerstr. 49, 79104 Freiburg, Germany
[2] Spemann Graduate School of Biology and Medicine (SGBM), University of Freiburg, Albertstr. 19a, 79104 Freiburg, Germany
[3] Faculty of Biology, University of Freiburg, Schänzlestr. 1, 79104 Freiburg, Germany
[4] Department of Medicine, Renal Division, Medical Center - University of Freiburg, Faculty of Medicine, University of Freiburg, Hugstetter Str. 55, 79106 Freiburg, Germany
[5] Laboratory for MEMS Applications, IMTEK - Department of Microsystems Engineering, University of Freiburg, Georges-Koehler-Allee 103, 79110 Freiburg, Germany
[6] III. Department of Medicine, University Medical Center Hamburg-Eppendorf, 20246 Hamburg, Germany
[7] BIOSS Centre of Biological Signalling Studies, University of Freiburg, Schänzlestr. 18, 79104 Freiburg, Germany
[8] Institute of Anatomy, University of Zurich, Winterthurerstr.190, 8057 Zurich, Switzerland

[+] Equal contribution

* Corresponding author:
Prof. Dr. Bernd Kammerer
Email: bernd.kammerer@zbsa.uni-freiburg.de
Center for Biological Systems Analysis
Albert-Ludwigs-University Freiburg
Habsburgerstraße 49
79104 Freiburg i. Br.
Germany
Tel: 0761-203-97137
Fax: 0761-203-97177

Supplementary Table 1: Primers used in this study for qPCR analyses.

Gene	Accession number		Sequence	Source
mTBP	NM_013684	FP	5’-CCCCTTGTACCCTTCACCAAT-3’	
		RP	5’-GAAGCTGCGGTACAATTCCAG-3’	[44]
mHk1	NM_001146100	FP	5’-GAAAGGAGACCAACAGCAGAGC-3’	
		RP	5’-TTCGTTCCTCCGAGATCCAAGG-3’	
mSord	NM_146126	FP	5’-GGATGGTCACTTTGCTTGTGGC-3’	
		RP	5’-GGTCTCTTTGCCAACCTGGATG-3’	
mAkr1b3	NM_009658	FP	5’-GGTGAAAGGAGCCTTCCAGAAG-3’	
		RP	5’-ATCCAGTGGGAAATAGTCGGGC-3’	
hHK1	NM_000188.2	FP	5’-CTGCTGGTGAAAATCCGTAGTGG-3’	
		RP	5’-GTCCAAGAAGTCAGAGATGCAGG-3’	[45]
hAKR1B1	NM_001628	FP	5’-CCAACTTCAACCATCTCCAGGTG-3’	
		RP	5’-GTCACCACGATGCCTTTGGACT-3’	
hSORD	NM_003104	FP	5’-GCCGATACAATCTGTCACCTTCC-3’	
		RP	5’-CGCCTTCCTCAAAGGTGACATTG-3’	
hGAPDH	NM_001256799.2	FP	5’-GTCTCCTCTGACTTCAACAGCG-3’	
		RP	5’-ACCACCCTGTTGCTGTAGCCAA-3’	[46]
hHSPCB	NM_001271969	FP	5’-CTCTGTCAGAGTATGTTTCTCGC-3’	
		RP	5’-GTTTCCGCACTCGCTCCACAAA-3’	

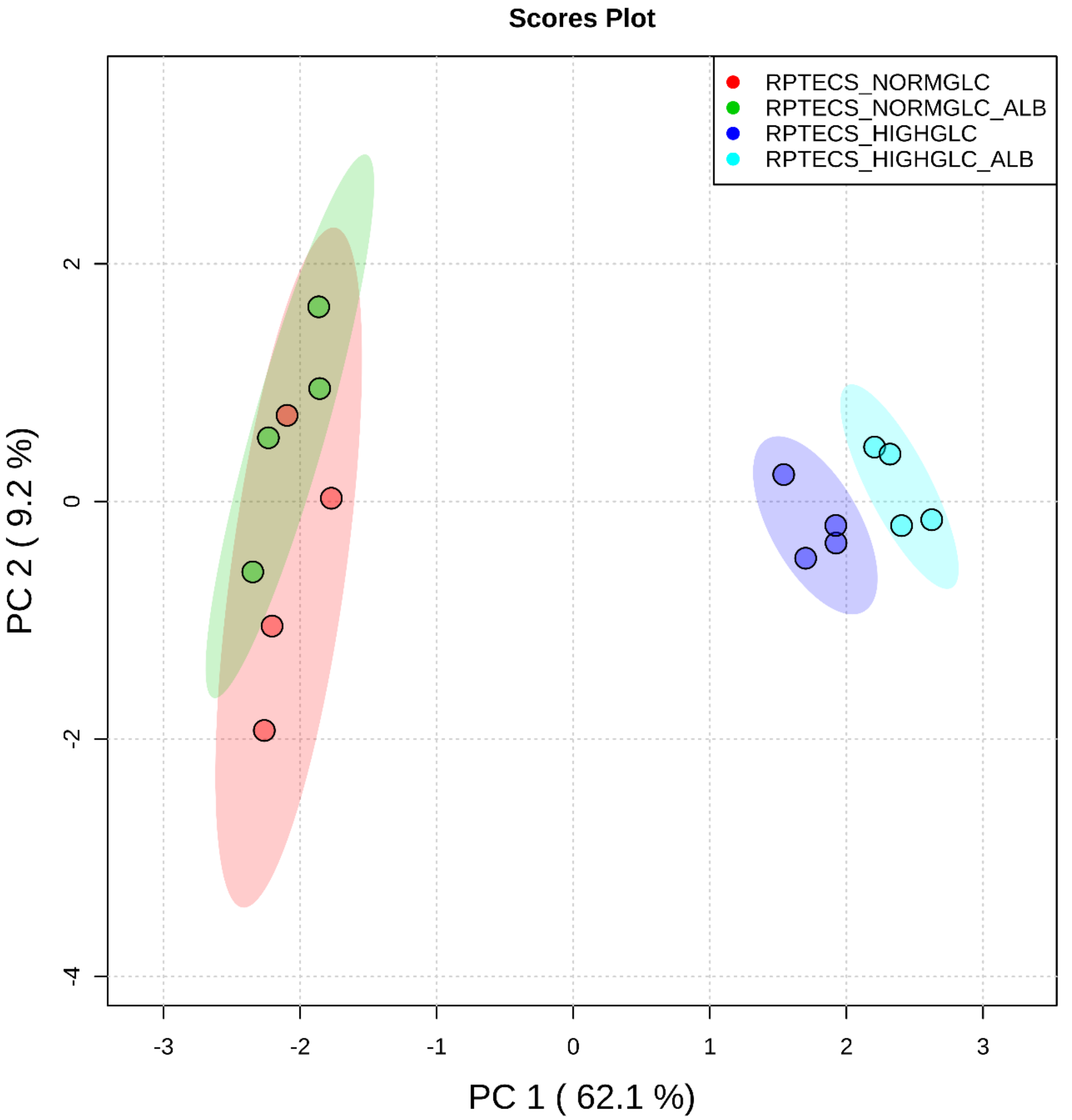

Supplementary Figure 1: Principal component analysis of RPTECs. PC2 reflected the biological variance and was not affected by glucose or albumin concentration.

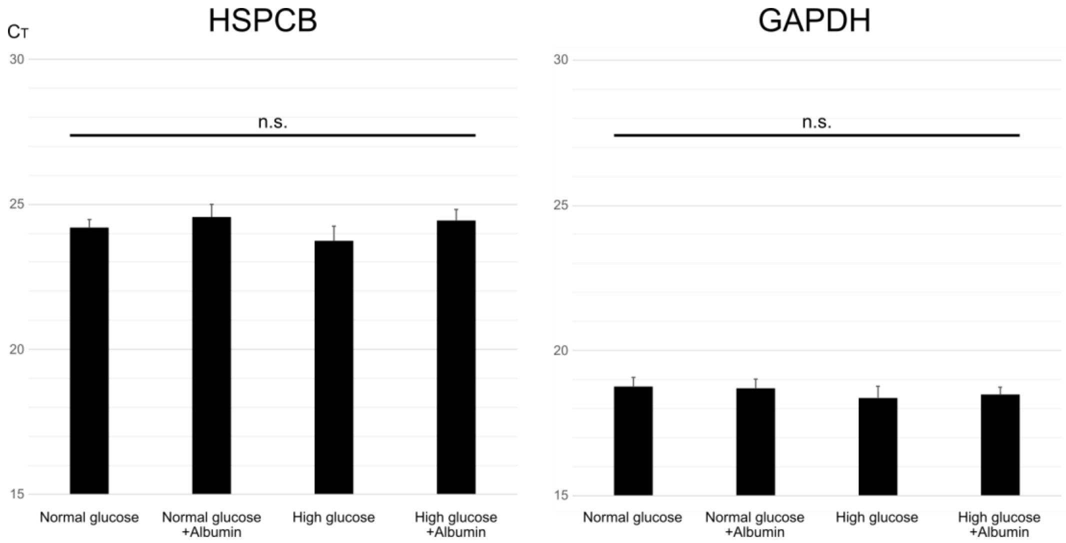

Supplementary Figure 2: Ct values of housekeeping genes used for quantitative RT-PCR in RPTECs. Expression levels of both housekeeping genes used for this study are not dependent on glucose or albumin concentrations. Columns display mean Ct- values of three replicates with 95% confidence interval (error bars). Ct – cycle threshold; HSPCB – heat shock 90kDa protein 1, beta; GAPDH – glyceraldehyde-3-phosphate dehydrogenase; n.s. – not significant.

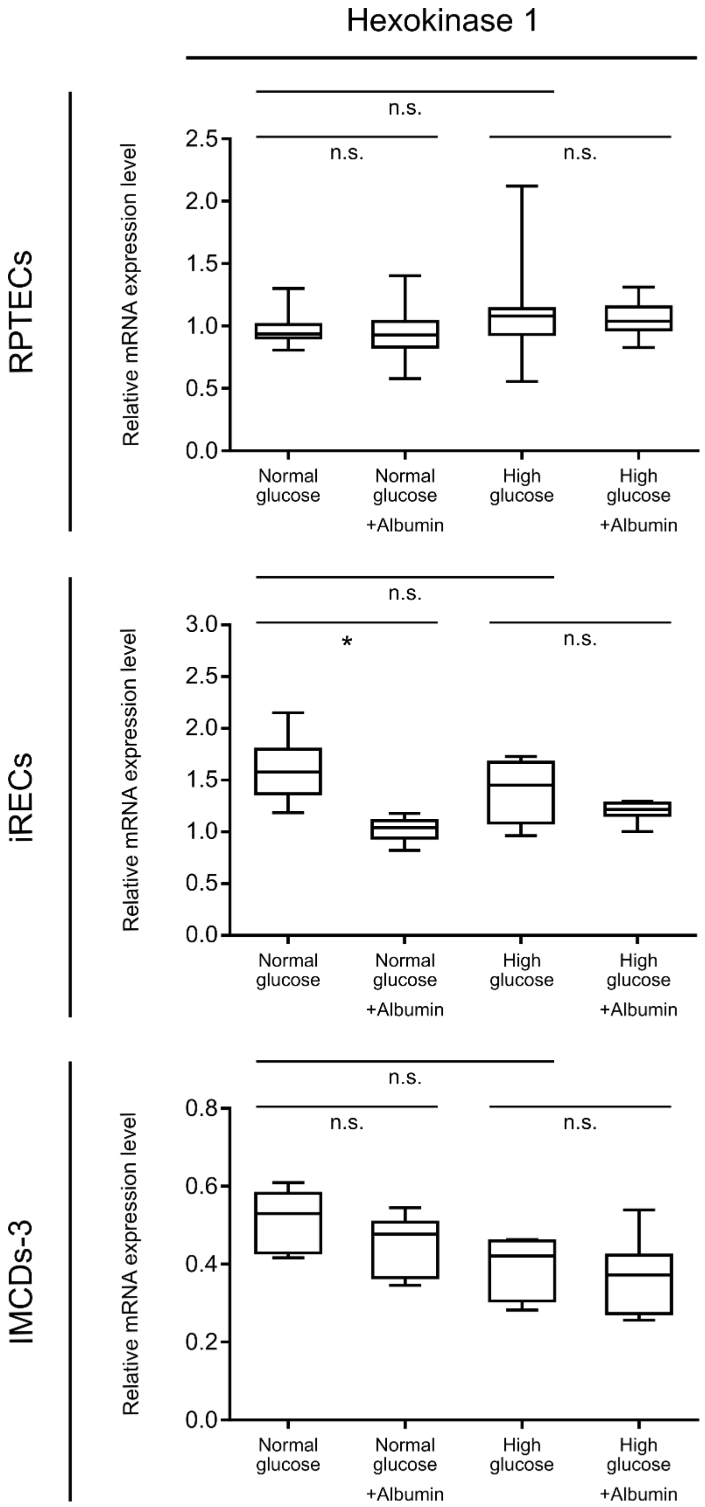

Supplementary Figure 3 : Relative mRNA expression level of hexokinase 1 as detected by qPCR analysis. Comparison between normal and high glucose, high glucose w/wo albumin overload and normal glucose w/wo albumin overload. Data are shown for RPTECs (upper line), iRECs (intermediate line) and IMCD-3 (lower line). Boxplots display mean values of three replicates with 95% confidence interval (whiskers). p-values: n.s. not significant, * <0.05.

References

44. Ferreira MJ, McKenna LB, Zhang J, Reichert M, Bakir B, Buza EL et al. Spontaneous Pancreatitis Caused by Tissue-Specific Gene Ablation of Hhex in Mice. Cell Mol Gastroenterol Hepatol 2015;1(5):550–69.

45. Hariton F, Xue M, Rabbani N, Fowler M, Thornalley PJ. Sulforaphane Delays Fibroblast Senescence by Curbing Cellular Glucose Uptake, Increased Glycolysis, and Oxidative Damage. Oxid Med Cell Longev 2018;2018:5642148.

46. Izumi-Nakaseko H, Kanda Y, Nakamura Y, Hagiwara-Nagasawa M, Wada T, Ando K et al. Development of correction formula for field potential duration of human induced pluripotent stem cell-derived cardiomyocytes sheets. Journal of Pharmacological Sciences 2017;135(1):44–50.
